# Explaining juvenile idiopathic arthritis to paediatric patients using illustrations and easy-to-read texts: improvement of disease knowledge and adherence to treatment

**DOI:** 10.1186/s12969-021-00644-9

**Published:** 2021-11-08

**Authors:** Christiane Reiser, Nina A. Zeltner, Beatrix Rettenbacher, Petra Baumgaertner, Martina Huemer, Christian Huemer

**Affiliations:** 1 Department of Paediatrics, LKH Bregenz, Bregenz, Austria; 2 Department of Psychosomatics and Psychiatry, University Children’s Hospital Zurich, Zurich, Switzerland; 3 RWU Hochschule Ravensburg-Weingarten, University of Applied Sciences, Weingarten, Germany; 4 Division of Metabolism and Children’s Research Center, University Children’s Hospital, Zurich, Switzerland

## Abstract

**Introduction:**

Juvenile idiopathic arthritis (JIA) is the leading chronic rheumatic disease in childhood. To achieve adherence to therapy, in-depth understanding of disease and treatment options are important.

**Objective:**

Development of specifically designed illustrations and standardised, easy-to-read texts for children and adolescents with JIA. Education materials were tested for comprehensibility and content validity. We hypothesised that children would be able to increase their knowledge about JIA after presentation of materials.

**Methods:**

The illustrations were designed by a graphic artist and the informative texts consecutively transformed to easy-to-read language. The materials appear as a modular system to allow individualized information for each patient.

The illustrations and texts were tested for knowledge gain and improvement of self-efficacy in children affected by JIA/ rheumatic diseases and controls. Health-related quality of life (HRQoL) was tested as an overall assessment of patients’ well-being.

**Results:**

46 controls (71% female) and 38 patients (48% female) with a median age of 11 years were tested in a standardised setting. In both groups knowledge gain was significant (controls: *t* (44) = 11.08, *p* < 0.001, *d* = 1.65; patients: *t* (37) = 7.48, *p* < 0.001, *d* = 1.21). The control group had a significantly higher enhancement of disease knowledge compared to patients’ group (*p* = .046) The follow-up testing was only performed in one school class (20 controls) due to Covid-19 pandemic with significant improvement compared to the pre-test results (*p* = .002).

The enhancement of self-efficacy through the teaching session was significantly higher in the patients’ group. No impairment of HRQoL was seen.

**Conclusion:**

Explaining juvenile rheumatic diseases and therapeutic strategies is an important task in paediatric rheumatology. To avoid incomprehensible explanations in medical jargon, illustrations and easy-to-read texts were developed. Standardised presentation of the newly created materials resulted in a significant improvement of disease knowledge in patients and controls in addition to an enhancement of self-efficacy in patients.

## Background

Juvenile idiopathic arthritis (JIA) is the most frequent chronic rheumatic disease in childhood and may cause short- and long-term morbidity [1]. Classification criteria exist for seven different forms of JIA. By definition, children with chronic arthritis, younger than 16 years at disease onset with a disease duration exceeding 6 weeks are, after exclusion of other diagnoses, considered to have JIA [2]. JIA is an autoimmune disease that can involve different organ systems beyond the joints, e. g. the eyes. The disease course is heterogeneous and varies from monophasic to relapsing disease activity. To avoid chronic joint damage and impairment of physical and social development, prompt initiation of medical and complementary treatment (e. g. physiotherapy or ergotherapy) is essential. Today, with effective drugs available, we are in the favourable position to treat these patients with encouraging results.

Since the 1990s, the perception of the value of patient education in chronic paediatric diseases has fundamentally changed. Patient education has become an important component of disease management and is addressed by the EULAR recommendations for the management of early arthritis in adulthood [3]. In an extended report, the EULAR recommendations for patient education postulate the necessity of theoretical disease and treatment knowledge [4].

Furthermore, nowadays, patients should be trained in health management to enable their participation in decisions regarding their health (shared-decision-making) [5].

The positive effects of patient education have been well documented. Patient education significantly improves disease knowledge, promotes health behaviour [4, 6] and empowers patients to manage their lives with the disease and improve their health-status [4]. Several studies showed that the impact of patient education on health-related behaviour can be marginal or short-lived due to lack of long-term investigation of patients’ perception [7, 8]. However, if patient education is combined with interventions targeting and enhancing self-efficacy, long-term changes of health-related behaviour as well as health-related quality of life can be achieved [5, 9–12].

Pictorial information can be helpful for young patients and for individuals with low (health) literacy [13], because generally, pictures can be remembered more easily than words (“pictorial superiority effect”) [14]. When illustrations are combined with easy- to- read texts, a significant knowledge gain and simplified recalling can be observed [14].

In order to meet these requirements for educational materials, we created illustrations in combination with easy-to-read texts to teach JIA patients and their families (usually the parents and caregivers) the basics of their autoimmune disease, the JIA subtypes and therapeutic options. To our knowledge, no other evaluation of this kind of paediatric educational materials has so far been performed in JIA patients.

## Methods

### Development of educational materials

Twenty-nine illustrations were designed by a professional graphic artist (BR) after detailed introduction to the topic by paediatric rheumatologists (CR, CH), who also wrote the informative texts. These texts were consecutively transformed to easy – to – read language by qualified “easy language” specialists (Büro für Leichte Sprache. Lebenshilfe Bremen e. V. www.leichte-sprache.de) to achieve comprehensibility. In this process, the original German text is “translated” into “easy language”, a text form following special rules like simple wording or use of short sentences without complex grammatical constructions. “Easy language” has been developed for persons with disabilities, dyslexia, non-mother-tongue speaker, etc. to facilitate the understanding of all kind of texts and issues [15].

### Structure of educational materials

**Fig. 1:**
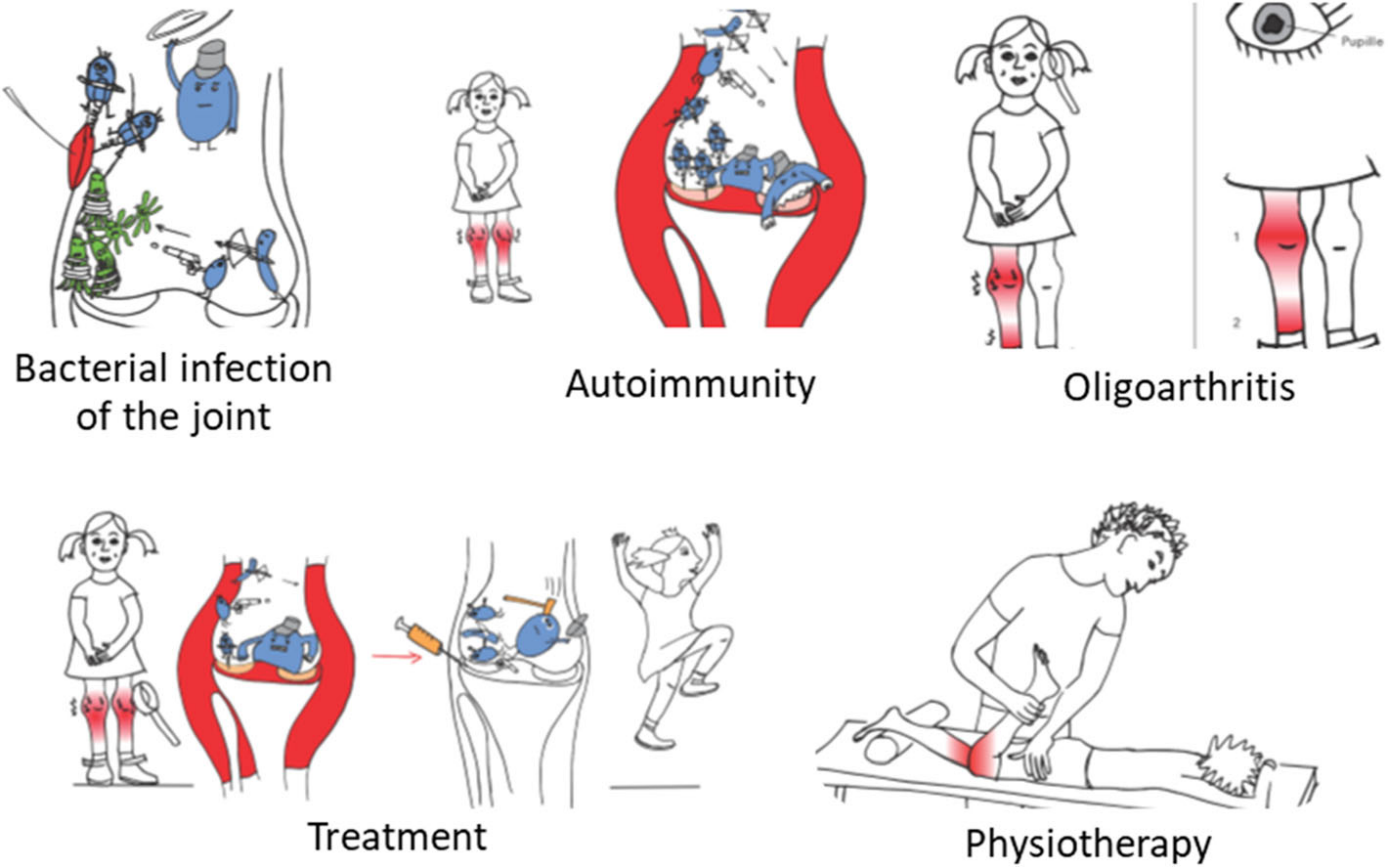
Examples of the illustration used in the study. The texts (not shown) in the illustration are German. A selection of Illustrations and texts as used the study protocol are provided in the supplement

**Fig. 2:**
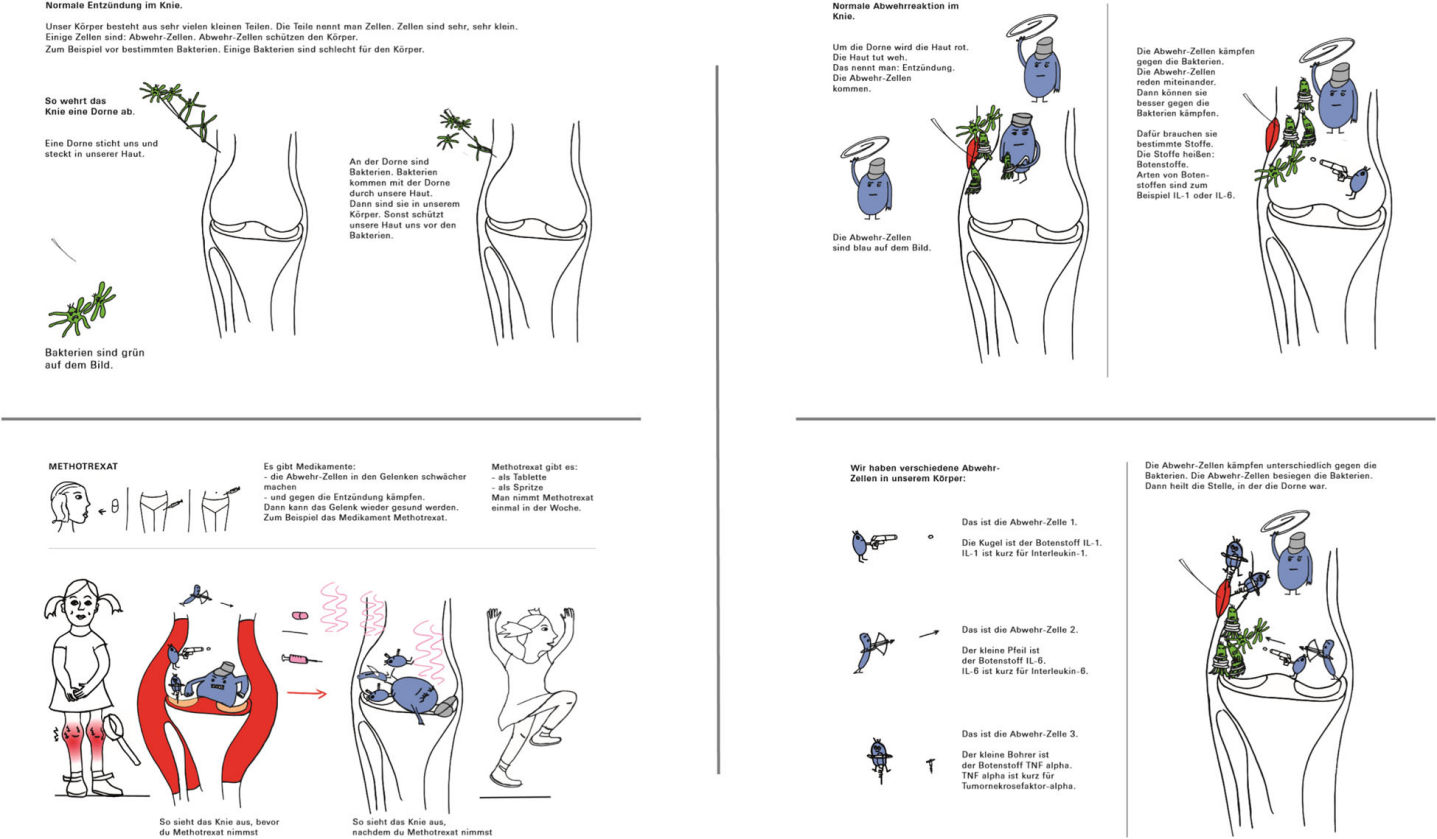
Selection of educational material used in the study protocol (texts are in German) For the whole package please contact the authors

Topics addressed in the illustrations are listed in Table 1. The educational materials were designed as a modular system allowing health care specialists to select the individual information needed for each patient. A representative selection of illustrations was chosen for the validation process consisting of illustrations for bacterial infection, autoimmunity, oligoarthritis (JIA subtype), treatment with ibuprofen/methotrexate/biologicals and physiotherapy (graphics see Fig. 1; selection of educational materials as used in the study protocol see Fig. 2).

**Table 1:**
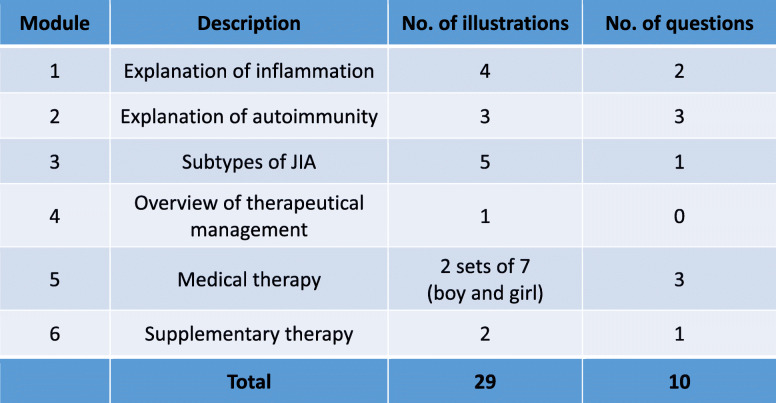
Overview of the modular structure of educational materials and weighting in the questionnaire (No.: number, JIA: Juvenile idiopathic arthritis)

### Participants and test setting

**Fig. 3:**
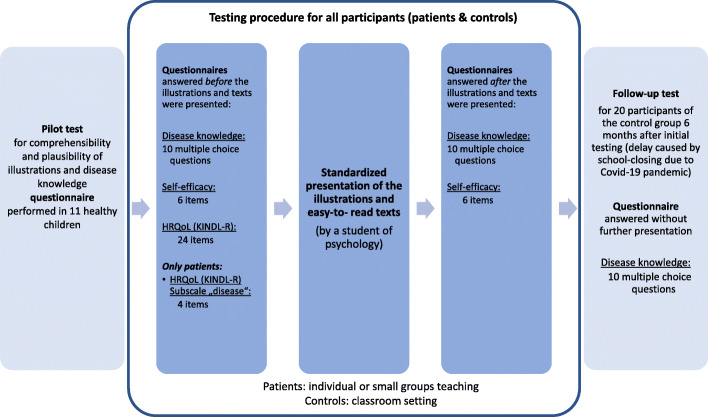
Testing procedure performed. HRQoL: Health related quality of life; KINDL-R; questionnaire for HRQoL in children (German: KINDL-R), revised revision

**Fig. 4:**
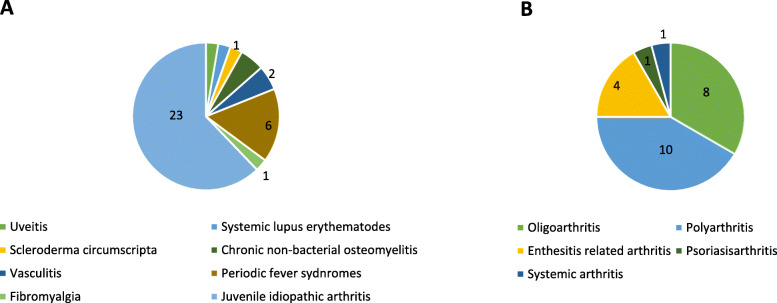
A: Patient characteristics: diagnoses (*n* = 37). B. Patient characteristics: numbers of patients with different subtypes of juvenile idiopathic arthritis (*n* = 23)

For the patients’ group 38 children (different paediatric rheumatic diseases or JIA at all stages of disease) being followed in the paediatric rheumatology clinic of Bregenz Hospital, were asked to participate in the study during their scheduled visit. The children received a standardised presentation (reading of the texts) of the selected illustrations by one student of psychology (PB). The procedure of the testing including the questionnaires used for the assessment of disease knowledge and psychological constructs is shown in Fig. 3. The duration of the testing was approximately 15–35 min depending on individual assistance needed. A detailed description of all participants is shown in Table 2. Figure 4 A and B show the details of patients’ diagnoses and subtypes of JIA.

**Table 2:**
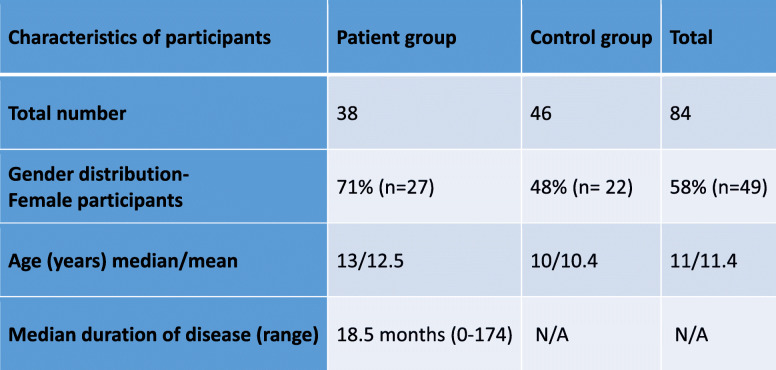
Characteristics of the study groups. N/A not applicable

The control group was composed of 46 children aged 7–15 years. Of those, 40 children not affected by JIA or any other rheumatic disease were recruited in fourth grade primary school and first grade secondary school classes in Bregenz, Austria. Six children were recruited in the Bregenz children’s hospital, where they were admitted for minor surgical or testing (e. g. allergy testing) interventions. Parents of the children recruited in schools had been informed by a letter about the aim of the study and had to sign a written informed consent prior to the testing. Parents of the children recruited in hospital were informed about the study and gave their oral informed consent. Children were invited to participate and completed the questionnaires. Schoolchildren were instructed as a class with a group size of approximately 20, the children in the hospital individually. The testing was performed as described above. Fluency in German and sufficient mental capability were presumed in all participants.

### Questionnaires

#### Knowledge gain

A multiple- choice questionnaire was developed consisting of 10 questions, each with three alternative answers. The questionnaire was evaluated in a pilot test by 11 healthy children aged 6 to 8 years without any relation to rheumatological topics to check for comprehensibility and plausibility, and proved to be feasible for the study cohort after minor adaptions of the wording of the questions. The results of this pilot group testing are not included in this manuscript.

The weighting of the modules in the questionnaire can be seen in Table 1. Knowledge gain was quantified by the difference between percentage of correct answers before and after the standardised presentation.

The planned follow-up testing 6 weeks later unfortunately had to be cancelled due to closing of schools caused by the Covid-19 pandemic. In one school class (*n* = 20) the testing was performed 23 weeks after the initial presentation (Fig. 3). The intended follow-up testing of patients could not be performed due to perturbed time intervals of patient visits, closing of all regular outpatient activities (only emergency visits were allowed) and restrictions of non-medical study staff to enter the hospital (PB).

#### Self-efficacy

Self-efficacy was assessed using a subscale (6 items) of the questionnaire published by Lohaus and Nussbeck assessing resources of children and youth [16]. Each question is answered on a 4 point Likert scale, high values suggest high self-efficacy [16]. The published questionnaire was validated in a large cohort of more than 2500 children. The subscale of self-efficacy showed good internal consistency with Cronbach’s *α* = .81. The questionnaire was answered before and after the presentation of the educational materials (Fig. 3).

#### Health-related quality of life

Health related quality of life (HRQoL) was tested using the German revised version of the KINDL questionnaire (KINDL-R) [17]. The KINDL-R consists of 24 questions covering 6 scales: physical and emotional well-being, sense of self-worth, family, friends and school. These scales can be added up to a sum score. Additionally, patients answered the supplementary sub-scale “disease” of the questionnaire. The questionnaire aims at assessing how the disease influenced HRQoL during the last 7 days on a 5-point Likert scale.

The questionnaire shows good internal consistency of the total score with Cronbach’s *α* = .84. The version for children aged 7–13 years was used for all patients and controls, since this age group was most represented in the sample. The questionnaire was answered by all participants prior to the presentation of the materials (Fig. 3).

### Statistical methods

Knowledge gain was calculated by comparing numbers of correct answers in pre- and post-test with paired sample t-tests. Similarly, changes in self-efficacy were analyzed by paired sample t-tests. HRQoL subscale and sum scores were compared to normal populations [17, 18] . Cohen’s d served as indicator for effect size. A significance level of *p* < 0.05 was defined as statistically significant. IBM SPSS Statistics, Version 25 (IBM Corp., IBM SPSS Statistics for Windows) was used for statistical analyses.

All procedures in the study were conducted in accordance with the Helsinki Declaration (2000), the protocol was presented to the regional Ethics Committee, but needed no formal approval (EK-0.04-359). Informed consent was obtained from all participants / their legal representatives to be included in the study.

## Results

A detailed description of the cohort is displayed in Table 2. For the presentation of pre-existing rheumatic diagnoses in the patient group see Fig. 4A and B.

### Knowledge gain

**Fig. 5:**
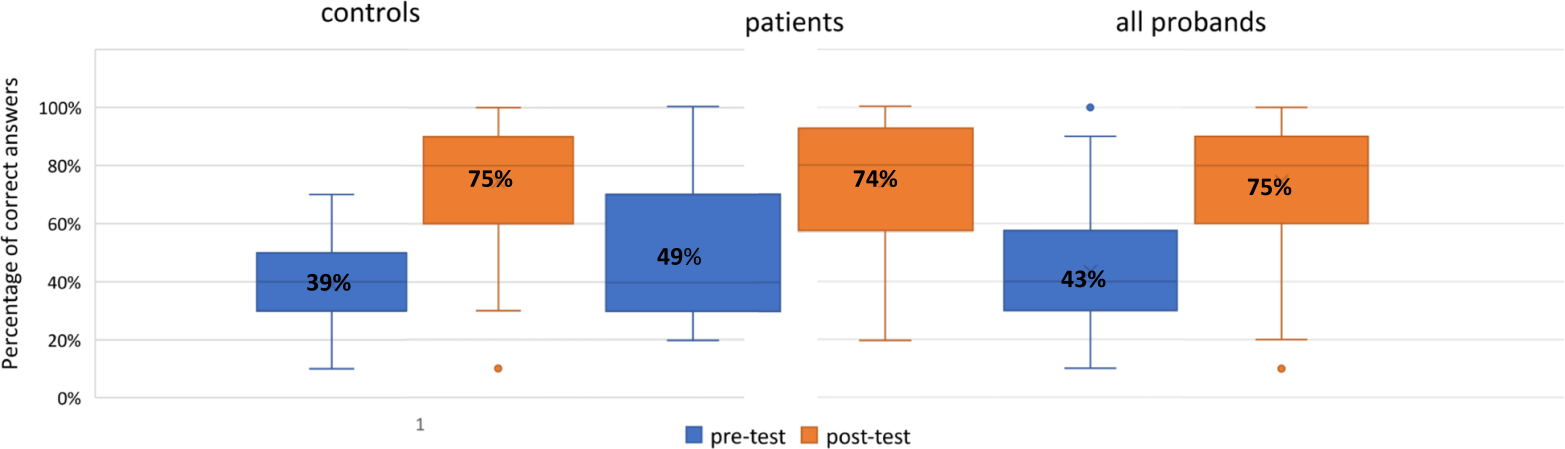
Knowledge gain in controls, patients and the complete cohort. Bold numbers in the boxes indicating median percentage of correct answers, whiskers indicating first (low) and third (high) quartile. Dots indicate outliers

The analysis of the questionnaire showed a significant knowledge gain in controls and in patients comparing correct answers in pre- and post-test results (patients: *M (SD)* pre-test = 4.87(2.18), *M (SD)* post-test = 7.45(2.35), *p* < .001, *d* = 1.21; controls: *M (SD)* pre-test = 3.89(1.50), *M (SD)* post-test = 7.42(2.35), *p* < .001, *d* = 1.65) (see Fig. 5).

In the control group the enhancement of disease knowledge was significantly higher (*M (SD)* = 3.53(2.14)) compared to the patients’ group (*M (SD)* = 2.58(2.13), *p* = .046) (testing was performed directly after presentation).

Due to the lock-down caused by the Covid-19 pandemic only 20 controls could be tested 23 weeks after the initial presentation. In this subgroup the participants showed a significant decline of knowledge compared to the post-testing (*M (SD)* = 9.3(1.13); *p* < .001). Still, a significantly improved result compared to the pre-test results (*M (SD)* = 4.5/1.64) was achieved in the follow-up testing (*M (SD)* = 6.7/1.66, *p* = .002).

### Self-efficacy

A significant enhancement of self-efficacy was observed in the patients group comparing pre- and post-testing results (*M (SD)* = 0.31(0.71); *p* = .007). No significant changes could be observed in the control group (*M (SD)* = 0.07(1.2); *p* = .357).

### HRQoL

No statistically significant difference to norms was found in scores on the six dimensions of physical and emotional well-being, sense of self-worth and well-being concerning family, friends and school in controls or patients (Table 3). Patients, who answered the “disease” subscale, were found to have significantly better scores compared to the published cohort (1050 children in rehabilitation centers with chronic diseases) for all age-groups analysed (females < 14 years: *t* (12) = 2.22, *M (SD)* = 75(23.51), *p* = 0.047, *d* = 0.61; females > 14 years: *t* (12) = 5.81, *M (SD)* = 85.9(16.01); *p* < .001, *d* = 1.61, males < 14 years: *t* (7) = 3.02, M (SD) = 81.77(16.51); *p* = .02, *d* = 1.07, males > 14 years: not analysed due to small sample size of two patients) [18].

**Table 3:**
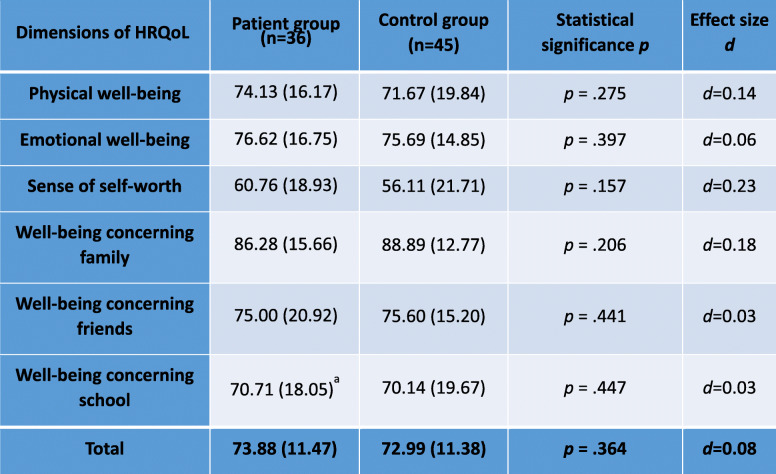
The KINDL-R questionnaire uses six dimensions of health related quality of life (HRQoL). No significant difference between the two groups regarding HRQoL was found. ^a^ *n*=35

## Discussion

In daily routine, health professionals face the problem that medical information is difficult to understand for patients. Only a small part of the facts provided by the medical team will be remembered [19]. Additionally, it is still common practice to use medical terms that are hard to understand, especially for children [12, 20]. Written health information provided by medical institutions is often inappropriate for the average literacy level of patients [21]. Thus, the patient is often rather confused than enlightened by standard materials.

In our study, we focussed on the development of educational materials that might contribute to a better understanding of specific rheumatic diseases (JIA and subtypes) and their treatment.

A systematic review [20] analysed the effect of pictorial information on health behaviour and disease-specific outcomes such as adherence to therapy or healthy eating habits. 54 randomized controlled studies analysing the effect of pictorial health information on patient’s behaviour and outcome were included in the review. A subgroup analysis explored the effects of pictures on patients with low (health) literacy. The authors found an increase of knowledge and understanding, when pictorial health information was presented, confirming previous studies [22, 23]- but no improvement of adherence was achieved [20]. However, in subgroup analyses, an improvement of dosing accuracy or medication concordance in patients with low health literacy was detected. The effectiveness of pictures was even higher when only a small number of words accompanied the illustrations [20].

Processing fluency describes the feeling of ease with which people handle information presented to them [11]. The effect of educational materials can be enhanced by the style of presentation [11]. If materials are of high processing fluency, readers develop a more positive perception of and attitude towards its content [11]. Our materials were developed to generate this processing fluency using perceptual fluency by high contrast to the background of the pictures, easy-to-understand language for linguistic fluency and limited mass of information for better retrieval fluency in order to generate a positive perception resulting in an improvement of health behaviour and health management [11, 24].

In this analysis, we were able to show a significant knowledge gain in a heterogeneous group of participants. In patients with chronic diseases adequate health education is a powerful tool to achieve treatment adherence and health management [12]. The comparison between the two groups showed a more pronounced enhancement of knowledge in the control group of rheumatic disease-naïve individuals compared to the patients who are in rheumatological care and therefore are already pre-educated.

The long-term effects of the educational materials were originally planned to be analysed 6–8 weeks after the initial teaching. We were able to perform the initial teaching of the classes on the last days of open schools prior to the lockdown due to the corona pandemic, but extended long-term analyses were impossible. After re-opening of schools and the start of the new school year we got the opportunity for follow-up testing in one fourth of the control participants (one class). Although the test results did not reach the levels of the post- teaching tests, we still could demonstrate that after almost half a year the children could recall a significant part of the information provided. In this setting, the students had no further contact to rheumatic diseases and additionally had to handle homeschooling and lock-down settings. But still, encouraging results were documented. We conclude that the presentation of the illustrations in combination with the accompanying text is a very effective way of long-lasting knowledge transfer.

Additionally, we investigated the impact of our illustrations on self-documented self-efficacy before and after presentation of the materials.

Self-efficacy is an important psychological target to change behavior successfully [25, 26]. The importance of self-efficacy for changes of health – related behaviour is well known [10]. We were unable to investigate behavioural changes- as most of the data was collected cross-sectionally, but we were able to demonstrate an enhancement of self-efficacy following the educational sessions. This is a remarkable effect, because health education that improves self-efficacy is effective in changing unfavorable health behaviour [10].

To get an impression of the burden of disease in our cohort HRQoL was investigated. No significant difference was observed concerning HRQoL between patients and controls. Large studies investigating this issue provided similar results. The German inception cohort study of newly diagnosed patients with JIA (ICON, including 953 patients and 491 healthy peers) showed the similarity of HRQoL of JIA patients compared to healthy peers during the disease course [27] and attributed this finding to optimized therapeutic conditions for the patients. In contrast, our cohort is very heterogeneous (a variety of rheumatic diagnoses, variable duration of disease, etc.) and therefore not completely comparable to the ICON study. Notably, in the subscale “disease” the patient group showed better results than the published reference cohort. In the reference cohort, children were suffering from chronic diseases such as atopic eczema, obesity or asthma and were tested during their hospitalisation in rehabilitation centers. In contrast, our cohort was in a generally good health condition (out-patient setting, planned appointment).

After complementation of the validation process, our educational materials were transformed to video sequences. The videos are available (in English and German) on a newly created website http://www.kinder-rheuma-info.com. The website is mainly in German but will be consecutively updated and translated to other languages.

### Limitations of the study

To evaluate self-efficacy the participants used a questionnaire before and after the teaching session. We did not investigate behavioural changes or outcome parameters in the patients’ group. After implementation of the educational materials in daily routine, follow-up investigations are needed to assess long-term adherence and improvement of health/disease behaviour.

## Conclusion

Our aim was to create illustrations and texts suitable for school age children that can be used easily and in a reasonable time during an outpatient visit to explain the etiology of rheumatic diseases, subtypes of JIA, the medication and the general treatment strategy.

Our illustrations and texts increased disease and treatment knowledge significantly in controls and patients. The significant enhancement of self-rated self-efficacy after the teaching suggests that our materials may induce behavioural changes such as improved adherence to treatment. This important aspect should be addressed in future research.

## Data Availability

The datasets used and analysed during the current study are available from the corresponding author on reasonable request.

## Abbreviations

EULAR: European League Against Rheumatism
HRQoL: Health related quality of life
ICON: German Inception Cohort of Newly diagnosed patients with JIA
JIA: Juvenile idiopathic arthritis
KINDL-R: Questionnaire for HRQo*L* in children (German: *KINDL-R*), *r*evised version
PRINTO: Paediatric Rheumatology International Trials Organisation
RCT: Randomized controlled trial
